# Pericardial- Rather than Intramyocardial Fat Is Independently Associated with Left Ventricular Systolic Heart Function in Metabolically Healthy Humans

**DOI:** 10.1371/journal.pone.0151301

**Published:** 2016-03-11

**Authors:** Peter Wolf, Yvonne Winhofer, Sabina Smajis, Draženka Jankovic, Christian-Heinz Anderwald, Siegfried Trattnig, Anton Luger, Michael Krebs, Martin Krššák

**Affiliations:** 1 Medical University of Vienna, Department of Internal Medicine III, Division of Endocrinology and Metabolism, Vienna, Austria; 2 Wilhelminenspital, Department of Internal Medicine I, Division of Oncology, Hematology and Palliative Care, Vienna, Austria; 3 Medical University of Vienna, Department of Biomedical Imaging and Image-guided Therapy, Centre of Excellence—High Field MR, Vienna, Austria; 4 Metabolic Unit, Institute of Biomedical Engineering, National Research Council, Padova, Italy; 5 Medical Direction, Specialized Hospital Complex Agathenhof, A-9322 Micheldorf, Carinthia, Austria; GDC, GERMANY

## Abstract

**Background:**

Obesity is a major risk factor to develop heart failure, in part due to possible lipotoxic effects of increased intramyocardial (MYCL) and/or local or paracrine effects of pericardial (PERI) lipid accumulation. Recent evidence suggests that MYCL is highly dynamic and might rather be a surrogate marker for disturbed energy metabolism than the underlying cause of cardiac dysfunction. On the other hand, PERI might contribute directly by mechanic and paracrine effects. Therefore, we hypothesized that PERI rather than MYCL is associated with myocardial function.

**Methods:**

To avoid potential confounding of metabolic disease 31 metabolically healthy subjects (age: 29±10yrs; BMI: 23±3kg/m^2^) were investigated using ^1^H-magnetic resonance spectroscopy and imaging. MYCL and PERI, as well as systolic and diastolic left ventricular heart function were assessed. Additionally, anthropometric data and parameters of glucose and lipid metabolism were analyzed. Correlation analysis was performed using Pearson’s correlation coefficient. Linear regression model was used to show individual effects of PERI and MYCL on myocardial functional parameters.

**Results:**

Correlation analysis with parameters of systolic heart function revealed significant associations for PERI (Stroke Volume (SV): R = -0.513 p = 0.001; CardiacIndex (CI): R = -0.442 p = 0.014), but not for MYCL (SV: R = -0.233; p = 0.207; CI: R = -0.130; p = 0.484). No significant correlations were found for E/A ratio as a parameter of diastolic heart function. In multiple regression analysis CI was negatively predicted by PERI, whereas no impact of MYCL was observed in direct comparison.

**Conclusions:**

Cardiac fat depots impact left ventricular heart function in a metabolically healthy population. Direct comparison of different lipid stores revealed that PERI is a more important predictor than MYCL for altered myocardial function.

## Background

Obesity and type 2 diabetes mellitus (T2DM) are both major risk factors for the development of cardiovascular disease and heart failure [1]. It has been suggested that intramyocardial (MYCL) and/or pericardial (PERI), i.e. the sum of epicardial + paracardial adipose tissue, might contribute to metabolic cardiomyopathy [2–4]. yc

Both, MYCL and PERI, are closely associated with disturbed glucose metabolism including impaired glucose tolerance [5] and T2DM [6, 7], resulting in functional as well as morphological changes of the heart: Increased MYCL content has been reported to be associated with decreased left ventricular (LV) diastolic functional parameters in lean healthy volunteers following short term caloric restriction [8] and was shown to be an independent predictor of diastolic dysfunction in diabetic patients [9]. With regard to PERI, there is a strong inverse association with LV diastolic function in men with metabolic syndrome [10]. Also in obese females hemodynamic systolic as well as diastolic parameters closely correlated with the amount of epicardial fat [11].

Our recent studies suggest that MYCL accumulation is not directly related to insulin resistance [6] and is highly dynamic, quickly responding to changes in the metabolic and hormonal milieu [12–14]. Furthermore, sufficient MYCL stores might be prerequisite to stimulated systolic function in response to catecholamine-induced stress [14]. This indicates that in humans MYCL content might rather be a surrogate marker for disturbed whole body glucose and lipid metabolism including hyperinsulinemia/hyperglycemia [12, 13] and altered availability of free fatty acids [14], rather than the underlying causal factor responsible for impaired myocardial function. On the other hand, PERI is a metabolically active, visceral, thoracic fat depot, which shares myocardial blood supply and might contribute directly to cardiac lipotoxicity by secreting proinflammatory adipokines [15]. Therefore, we hypothesized that PERI rather than MYCL is associated with myocardial function.

To avoid potential confounding of metabolic disease we analyzed the associations of MYCL and PERI on parameters of left ventricular heart function in a slightly overweight, but metabolically healthy population. We hypothesized that in case of a relevant influence of intra- and/or pericardial steatosis even subtle changes in cardiac lipid stores are associated with altered left ventricular function.

## Materials and Methods

All data of cardiac magnetic resonance (MR) spectroscopy and imaging measurements performed at the MR Centre of excellence at the Medical University of Vienna from 2010 to 2015 was analyzed. Data were collected and pooled from five clinical experimental studies. Data of MYCL of all subjects included in this analysis was published previously [13, 14, 16–18]. During the present data of PERI and left ventricular diastolic function was assessed in all subjects. Of note, only metabolically healthy individuals that participated in these studies as parts of the healthy control groups were included and no patient with known endocrine disorder was included. All MR measurements and blood tests were performed for research purpose only. Exclusion criteria were the presence of impaired fasting glucose, type 2 diabetes mellitus, body mass index (BMI) ≥ 30 kg/m^2^, dyslipidemia, elevated liver enzymes, regular intake of medication, any acute or chronic inflammatory disease, arterial hypertension, evidence of coronary artery disease, as well as general contraindications to perform MR measurements like a tendency towards claustrophobia or metal devices.

The ethical committee of the Medical University of Vienna approved performing the experiments and written informed consent was obtained from all participating subjects.

Subjects were investigated under resting conditions in the morning after an overnight fast of at least ten hours and were asked to refrain from intensive physical training, to stop regular moderate exercise and to ingest an isocaloric diet (30 kcal/kg/day, carbohydrate/protein/fat: 55%/15%/30%) for three days prior to the MR measurements.

**Myocardial lipid content** (**MYCL**) was measured by ECG-gated single voxel localized ^1^H-MRS as previously described [6, 13, 14, 16, 18]. Volume of interest was positioned in the interventricular septum to avoid signal alterations by epicardial fat. PRESS sequence (typical VOI = 15×10×30 mm^3^; TE = 30 ms; NA = 2x4 for water signal; 2 x 8 for water suppressed signal) data acquisition was performed during multiple repeated breath holds. MYCL was calculated from the ratio of summed area of methylene and methyl groups to that of water following the T_1_ and T_2_ relaxation correction based on relaxation times of skeletal muscle [19] as percent of tissue water MRS signal. For the **assessment of left ventricular function by MR imaging** cardiac MR visualization and analyses ARGUS software (Siemens AG Healthcare, Erlangen, Germany) was used. Visualization of cardiac function was performed using prospective ECG-gated cine true fast imaging with steady-state precession (TrueFISP) sequences in 2-chamber, 4-chamber and short axes orientation. Short axes images were used to quantify left ventricular global (end-diastolic and end-systolic volume, stroke volume, ejection fraction and myocardial mass) via ARGUS software (Siemens, Erlangen, Germany). These volumetric parameters were normalized to body surface area, using the Dubois-formula. Ejection fraction, stroke volume and cardiac index were used as primary indices of left ventricular systolic function. Additionally we determined left ventricular diastolic function using Flash-based (fast low angle shot) prospective ECG-gated cine phase contrast sequence and post-processing via ARGUS software to determine early (E) and late atrial (A) peak filling rates and the E/A ratio of mitral inflow. Deceleration time was assessed as the time from the maximum E point to baseline [18, 20–23]. Left ventricular diastolic function index (LVDI) was calculated as the ratio of early diastolic peak filling rate / end-diastolic left ventricular volume.

**Pericardial fat (PERI)** was assessed from T1 weighted ECG-gated cine true fast imaging with steady-state precession (TrueFISP) in four-chamber orientation in analogy to previously published methods [16, 17, 24]. Briefly, regions of interest were manually drawn along the borders of the fat surrounding the heart in three slices from the apex to the pulmonary trunk. The mean of the three slices is given in square centimeters. Due to the low amount of adipose tissue in lean, healthy subjects, a distinct separation between epicardial and pericardial fat was not possible. Therefore, the term pericardial fat, i.e. paracardial + epicardial adipose tissue, was used for our analysis.

Plasma **glucose, triglycerides, total cholesterol, HDL-cholesterol, LDL-cholesterol, GOT, GPT and GGT** was measured by routine laboratory methods at the Department of Laboratory Medicine, Medical University of Vienna (http://www.kimcl.at/). Plasma **FFA** concentration was measured in the laboratory of the Division of Endocrinology & Metabolism (micro-fluorimetric assay; WAKO, Neuss, Germany). Plasma **insulin** concentration was measured at the Department of Laboratory Medicine (http://www.kimcl.at/) or at the laboratory of the Division of Endocrinology & Metabolism (ELISA Mercodia, Sweden). Blood was drawn after an overnight fast of at least ten hours in the morning of each study day in close temporal relationship to the MR measurements.

Exploratory **statistical analysis** was performed using SPSS Version 23 (SPSSInc, Chicago, IL, USA) and Prism (GraphPad Software Inc, La Jolla, CA) Version 6 for Mac. Data is given as mean ± standard deviation. Correlation analysis was performed using Pearson’s correlation coefficient. Linear regression model was used to show individual effects of PERI and MYCL on myocardial functional parameters. Statistical significance was set at p < 0.05.

## Results

31 healthy subjects fulfilled inclusion criteria and were included in analysis. Anthropometric characteristics, laboratory parameters and data of MR measurements, including MYCL and PERI, as well as systolic and diastolic myocardial functional parameters are given in detail in Table 1. On average, included subjects were normal weight and all of them were metabolically healthy, indicated by blood levels of fasting plasma glucose, HbA1c, cholesterol and triglycerides within the normal range, as well as normal blood pressure.

**Table 1:** Anthropometric characteristics, laboratory parameters and data of magnetic resonance measurements; parameters of left ventricular (LV) myocardial function was normalized to body surface area using the Dubois-formula.

Sex (male/female)	19/12
Age (years)	29 ± 10
BMI (kg/m^2^)	23 ± 3
Fasting glucose (mg/dl)	85 ± 9
Insulin (μU/ml)	16 ± 6
Triglycerides (mg/dl)	106 ± 39
Total cholesterol (mg/dl)	183 ± 31
HDL cholesterol (mg/dl)	56 ± 13
LDL cholesterol (mg/dl)	106 ± 27
Free Fatty Acids (μmol/l)	413 ± 223
HOMA-IR	3.3 ± 1.2
HbA1c (%)	5.3 ± 0.3
GOT (U/l)	22 ± 5
GPT (U/l)	21 ± 9
GGT (U/l)	20 ± 7
BP systolic	123 ± 20
BP diastolic	80 ± 11
MYCL (%)	0.21 ± 0.15
PERI (cm^2^)	8.79 ± 7
Heart rate (bpm)	64 ± 7
Ejection fraction (%)	67 ± 6
Enddiastolic volume (ml/m^2^)	67 ± 18
Endsystolic volume (ml/m^2^)	23 ± 8
Stroke Volume (ml/ m^2^)	44 ± 12
Cardiac Index (ml/min/m^2^)	2.8 ± 0.8
Peak E (ml/s)	303 ± 112
Peak A (ml/s)	138 ± 54
E/A ratio	1.84 ± 0.6
LV diastolic index s^-1^	4.5 ± 1.7
Deceleration time (ms)	147 ± 50

**Fig 1:**
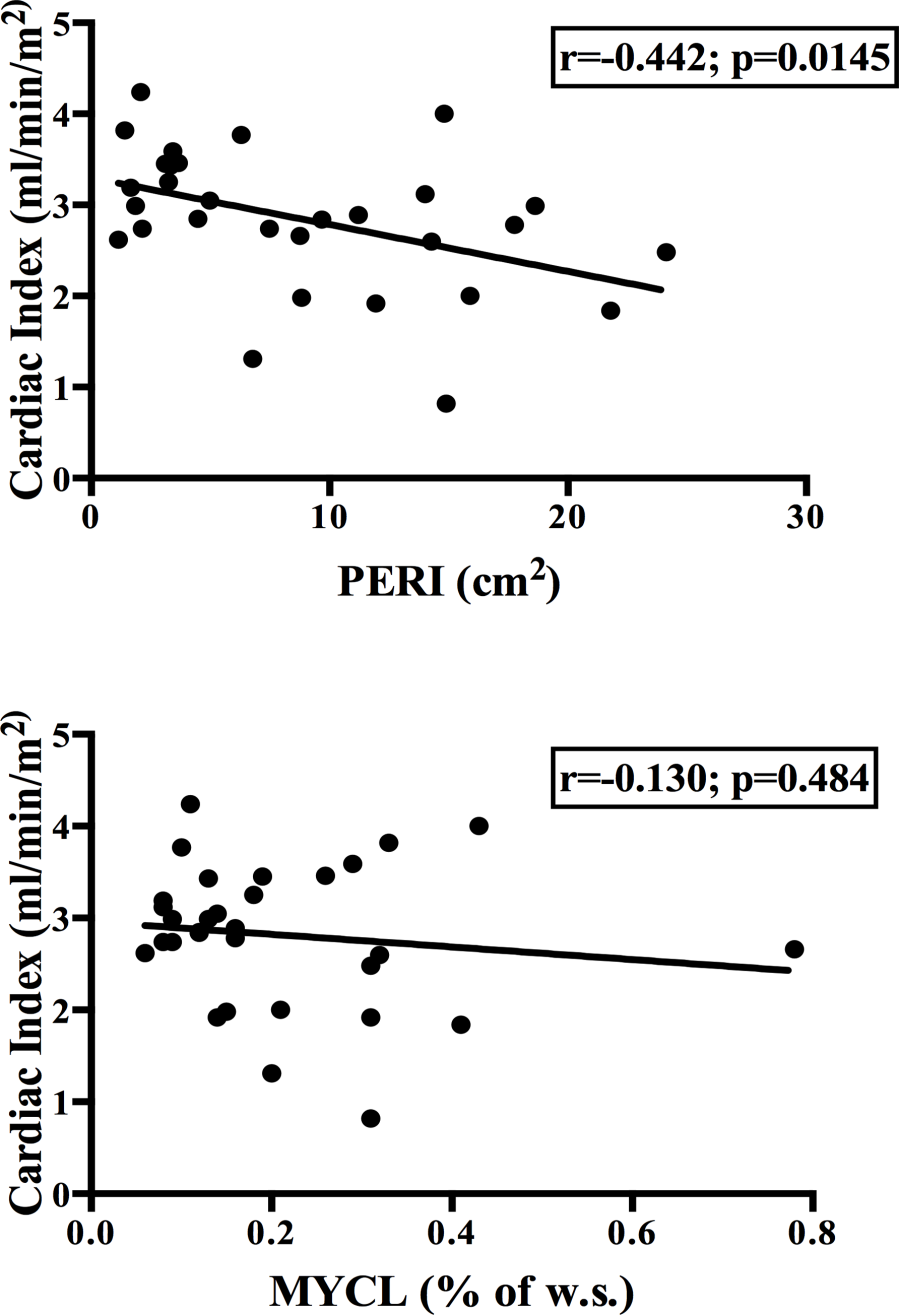
Correlation analyses of pericardial fat (PERI) and myocardial fat (MYCL) with cardiac index and E/A ratio in all subjects.

Correlation analysis revealed significant associations for PERI, but not for MYCL, with parameters of systolic heart function. Endsystolic and enddiastolic volume, stroke volume (SV) and cardiac index (CI) were strongly negatively related with PERI, whereas no significant correlation was found for MYCL. This indicates an impact of cardiac adiposity on systolic function even in a metabolically healthy population (see Fig 1).

With regard to diastolic heart function no relation could be found for both, MYCL and PERI, in a normal weight population. For a detailed list of data from correlation analysis see Table 2.

**Table 2:** R-values of pericardial (PERI) and intramyocardial (MYCL) adipose tissue with parameters of systolic and diastolic left ventricular functional parameters using Pearson correlation analysis in all subjects.

	PERI	MYCL
Heart Rate	0.151	0.272
Ejection fraction	0.115	0.008
Enddiastolic Volume	*-0*.*540*^#^	-0.235
Endsystolic Volume	*-0*.442*	-0.181
Stroke volume	*-0*.*513*^#^	-0.233
Cardiac Index	*-0*.442*	-0.130
Peak E	-0.206	-0.005
Peak A	0.065	0.285
E/A ratio	-0.247	-0.164
LV diastolic index	0.320	0.280
Deceleration time	-0.148	-193

* indicates p<0.05

^#^ indicates p<0.01

No relation could be found between metabolic parameters, i.e. plasma glucose, insulin, triglycerides and FFA concentrations, and cardiac fat depots, whereas PERI (R = 0.477; p = 0.008) strongly correlated with BMI. Additionally, there was a trend towards a mild association between PERI and MYCL (R = 0.333; p = 0.072).

With regard to metabolic parameters and heart function, fasting plasma glucose negatively correlated with ejection fraction (R = -0.490; p = 0.006) and CI (R = -0.416; p = 0.022), whereas no significant relation was found between TG, insulin, FFA, total cholesterol, and systolic and diastolic myocardial functional parameters.

Multiple regression analysis was performed, in order to evaluate the individual impact of MYCL, PERI, BMI and laboratory parameters on systolic heart function estimated by CI. CI was negatively predicted by HOMA and by PERI, whereas there was no impact of MYCL (see Table 3).

**Table 3:** Multiple regression analysis for cardiac index as depending variable; MYCL: myocardial lipid content.

*Cardiac Index*					
Independent variables:	R	E	β	T	p-value
MYCL	0.508	0.856	0.098	0.594	0.558
*PERI*	*-0*.*061*	*0*.*022*	*-0*.*525*	*-2*.*804*	*0*.010*
*HOMA*	*-0*.*313*	*0*.*112*	*-0*.*443*	*-2*.*809*	*0*.010*
FFA	-0.001	0.001	-0.306	-1.880	0.072
BMI	-0.012	0.043	-0.051	-0.278	0.783

PERI: pericardial fat content; HOMA: homeostatic model assessment index; FFA: free fatty acids; BMI: body mass index; R: regression coefficient, E: standard error

*indicates p<0.*05*

## Discussion

To the best of our knowledge, this is the first study demonstrating a predominant impact of PERI rather than MYCL on left ventricular myocardial functional parameters in a metabolically healthy population. Our results indicate that even in metabolically healthy subjects cardiac fat depots affect heart function, without being confounded by elevated circulating plasma substrate concentrations. This is of special interest, since the influence of circulating substrates or underlying metabolic disease on cardiac lipid stores is well established in animal models [25] as well as in humans [12–14]. Therefore we decided to include only metabolically healthy volunteers, to primarily elucidate the role of different cardiac fat depots as such.

Both, PERI and MYCL, are increased in obesity and T2DM and are associated with LV diastolic dysfunction. In uncomplicated T2DM, MYCL accumulation is augmented [7] and is associated with E/A deceleration as a marker of diastolic dysfunction independently of BMI, age, heart rate, visceral fat and blood pressure [9]. Moreover, increasing MYCL due to massive adipose tissue lipolysis induced by fasting is associated with a decrease in LV diastolic function in patients suffering from T2DM [26] and in healthy men [8]. This link between MYCL and cardiac dysfunction has been attributed to cardiac lipotoxicity secondary to lipid oversupply and an overstrain of FFA oxidation capacity, resulting in the accumulation of toxic intermediates of lipid metabolism and the development of LV hypertrophy, apoptosis and contractile dysfunction [27, 28].

On the other hand short-term changes in MYCL following acute interventions raise the possibility that myocardial lipid depots might also reflect a local, readily available source of energy to maintain heart function under conditions of reduced substrate availability, similar to skeletal muscle fat depots in trained athletes [29]. We have recently described that a constant availability of FFA is necessary for the acute catecholamine mediated response of the left ventricle to the stress of hypoglycemia even in healthy subjects [14]. In T2DM patients insufficiently controlled by oral medication, initiation of insulin therapy was followed by an 80% rise in MYCL, what might reflect the replenishment of depleted energy stores because of relative insulin deficiency [12]. Furthermore, an acute increase in MYCL even in the absence of plasma free fatty acids during a hyperglycemic-hyperinsulinemic clamp [13] was observed. In these studies the short-term increase in MYCL was not associated with impaired left ventricular function. Therefore, we suppose that MYCL might also have a protective effect by local substrate storage and to cope for acutely increased energy demands at least in healthy subjects, despite the well-known association with diastolic dysfunction in T2DM and obesity.

In contrast, evidence suggests that PERI exerts deleterious effects on myocardial function and therefore contributes to the development of heart failure in many ways. In this study PERI was assessed as the sum of epicardial and paracardial adipose tissue, since a distinct separation of different pericardial fat depots was not possible because of only little amounts of cardiac adipose tissue in lean volunteers. However, we assume that the majority of PERI in normal weight individuals is composed of epicardial adipose tissue.

Especially epicardial adipose tissue might reduce LV endsystolic- and enddiastolic volume mechanically in our group of healthy subjects by direct compression of the surrounding fat depots. Additionally, epicardial fat exerts paracrine effects on the myocardium, due to its anatomical proximity and direct contact, without being separated by any fascia, as well as because of sharing the same coronary blood supply [15]. This was underlined by recent studies by Greulich et al. in humans and animal models showing a link between adipokines secreted by epicardial fat and contractile dysfunction in cardiomyocytes [30].

Epicardial fat has been reported to be highly correlated with BMI, visceral fat and metabolic risk factors [5, 31], which is in line with our data, showing a significant association between PERI and BMI. Additionally, accumulation of PERI in patients suffering from metabolic syndrome and obesity was related with diastolic dysfunction [10, 11], but also correlated negatively with cardiac workload in obesity, as assessed by CI [32]. Our data emphasize that even in a metabolically healthy population, distinct changes in cardiac lipid depots exert relevant effects on heart function, since also in our study a strong significant, negative association of PERI with LV hemodynamic measurements, i.e. CI and SV, was observed and PERI negatively predicted CI in multiple regression analysis.

Metabolic diseases like severe obesity, metabolic syndrome and non-alcoholic fatty liver disease (NAFLD) are well known to be associated with impaired cardiac energy metabolism and function [33–35]. Therefore, a potential confounding of the underlying disease on parameters of heart function could not be ruled out in previous cross sectional studies. To avoid interference, we decided to include only metabolically healthy subjects, to primarily elucidate the role of different cardiac fat depots as such, without the confounders of elevated circulating plasma substrate concentrations and metabolic disease.

When the impact of MYCL and PERI on LV function was directly compared, multiple regression analysis revealed significant associations for PERI, whereas MYCL seems to play only a minor role in our group of metabolically healthy subjects. Of note, the lack of a significant correlation between MYCL and parameters of LV heart function might most likely be explained by the low amount of ectopic lipids in a metabolically healthy lean population. MYCL develops on the background of hyperglycemia and hyperinsulinemia [13] combined with increased FFA concentrations [25], a metabolic condition that is not present in insulin sensitive normal weight subjects. On the other hand, PERI extends over a much broader range compared to MYCL in healthy volunteers, wherefore associations can be detected earlier.

However, our findings are in line with recent data from obese men suffering from metabolic syndrome, in which only the accumulation of PERI, but not of MYCL, was linked to the severity of structural and functional alterations of the heart [10]. Also in non-diabetic men suffering from NAFLD parameters of LV diastolic function were negatively predicted only by hepatic lipid content and abdominal visceral fat mass and no impact of MYCL could be found [36]. Moreover, extensive weight loss following bariatric surgery was associated with a reduction of PERI accompanied by a distinct decrease in E/A ratio, while MYCL remained unchanged during the observation period [37]. Therefore we assume that PERI rather than MYCL might play the leading role in the development of heart failure in cardiac obesity, which is underlined by our findings even in a metabolically healthy population.

## Conclusions

Taken together, we clearly demonstrate an impact of cardiac fat depots on heart function in normal weight subjects. Our data suggest that even subtle changes in the diverse myocardial lipid stores significantly affect LV hemodynamics. Moreover, direct comparison of different fat depots revealed that PERI is a more important predictor than MYCL for altered systolic function in a lean, metabolically healthy population.
